# Neutrophil gene expression in COVID-19 patients with acute respiratory distress syndrome

**DOI:** 10.3389/fimmu.2025.1620745

**Published:** 2025-11-06

**Authors:** Hiroshi Ito, Masakazu Ishikawa, Jumpei Yoshimura, Yuchen Liu, Shuhei Sakakibara, Fuminori Sugihara, Hisatake Matsumoto, Haruhiko Hirata, Hiroshi Ogura, Jun Oda, Daisuke Okuzaki

**Affiliations:** 1 Department of Traumatology and Acute Critical Medicine, Osaka University Graduate School of Medicine, Suita, Osaka,, Japan; 2 Laboratory for Human Immunology (Single Cell Genomics), WPI (World Premier International Research Center Initiative) Immunology Frontier Research Center, Osaka University, Suita, Osaka, Japan; 3 Center for Infectious Disease Education and Research (CiDER), Osaka University, Osaka, Japan; 4 Laboratory of Immune Regulation, Immunology Frontier Research Center, Osaka University, Suita, Japan; 5 Core Instrumentation Facility, Immunology Frontier Research Center and Research Institute for Microbial Disease, Osaka University, Suita, Osaka, Japan; 6 Department of Respiratory Medicine and Clinical Immunology, Osaka University Graduate School of Medicine, Suita, Osaka, Japan; 7 Genome Information Research Center, Research Institute for Microbial Diseases, Osaka University, Suita, Osaka,, Japan; 8 Institute for Open and Transdisciplinary Research Initiatives, Osaka University, Osaka, Japan

**Keywords:** acute respiratory distress syndrome, coronavirus disease 2019 (COVID-19), neutrophil, single-cell RNA sequencing, gene expression

## Abstract

**Background:**

Although an increase in neutrophil count has been observed in patients with coronavirus disease 2019 (COVID-19), the relationship between the systemic neutrophil transcriptome and clinical course of COVID-19 remains unclear. Hence, we examined the relationship between the clinical course and RNA sequencing analysis results in COVID-19 patients.

**Methods:**

Peripheral blood samples were obtained from 28 patients with COVID-19-associated ARDS and 16 healthy controls. Bulk RNA sequencing was performed, and clustering analysis was used to explore relationships between gene expression and clinical characteristics. In a separate cohort, neutrophils were isolated from the peripheral blood of five COVID-19 patients with ARDS for single-cell RNA sequencing to further characterize the neutrophil subpopulations.

**Results:**

In bulk RNA sequencing analysis, COVID-19 patients with ARDS had elevated gene expression associated with neutrophils compared with healthy controls.Clustering analysis revealed no differences in the clinical characteristics of COVID-19 patients with ARDS. In the single-cell RNA sequencing analysis, clustering analysis showed that the patients were divided into two groups: those who could be weaned from the ventilator within 28 days and those who could not be weaned.

**Conclusion:**

These findings indicate that differences in neutrophil gene expression may have important clinical implications. This study may support the exploratory identification of genomic factors, such as neutrophil gene expression, that are relevant to clinical parameters.

## 1 Introduction

Coronavirus disease 2019 (COVID-19) is caused by severe acute respiratory syndrome coronavirus 2 (SARS-CoV-2) (1). COVID-19 was first reported in China in December 2019 (2), following which it rapidly spread worldwide and was declared a pandemic by the World Health Organization (WHO) in March 2020. Clinical observations have revealed a wide range of disease manifestations, ranging from asymptomatic cases to influenza-like symptoms, severe cases requiring mechanical ventilation, and in some cases, fatality. COVID-19 is characterized by respiratory symptoms; approximately 15% of patients develop pneumonia, and 5% develop severe conditions such as respiratory failure due to acute respiratory distress syndrome (ARDS), shock, or multi-organ failure (3). The median duration of mechanical ventilation is 12 days in cases requiring invasive mechanical ventilation (4). COVID-19 patients with ARDS, a serious condition, may require long-term mechanical ventilation. Therefore, accurate treatment selection during severe illness is clinically important.

Excessive neutrophil inflammation is considered a key factor contributing to lung injury in ARDS, and neutrophils are believed to play an important role in its pathophysiology (5). Alveolar macrophages secrete inflammatory cytokines that mobilize neutrophils and monocytes, thereby promoting inflammation. Activated neutrophils release reactive oxygen species and proteases (e.g., neutrophil elastase, myeloperoxidase, and matrix metalloproteinases) and contribute to damage via neutrophil extracellular trap (NET) formation (6). Disruption of epithelial and endothelial barriers leads to neutrophil infiltration into the interstitium and alveoli, which is believed to contribute to alveolar fibrosis and extended mechanical ventilation (7). The number of neutrophils in bronchoalveolar lavage fluid is relatively high in COVID-19 patients requiring mechanical ventilation (8–10), indicating the important role of local neutrophils in the lungs. The increased number of inflammatory monocytes and neutrophils suggests the importance of peripheral blood neutrophils (11), and signatures associated with neutrophil activation are significantly enriched in whole-blood transcriptomes of patients with severe disease manifestations (12). These neutrophil activation genes are highly expressed in specific subsets of lymphocytes and neutrophils, as suggested by the results of single-cell RNA sequencing (RNA-seq) (13). These findings suggest that neutrophil subtypes, based on their maturity, are associated with the disease state or clinical outcome.

However, studies examining the relationship between gene expression in peripheral blood cells during severe illness and subsequent clinical courses in COVID-19 patients with ARDS, such as ARDS alleviation and the possibility of early weaning from mechanical ventilation are limited. Therefore, we examined the relationship between the clinical course and results of whole-blood RNA-seq and single-cell RNA-seq analyses of neutrophils in COVID-19 patients.

## 2 Materials and methods

### 2.1 Study design and setting

This prospective, observational, single-center study was conducted at the Graduate School of Medicine, Osaka University. Approval was obtained from the Institutional Review Board of Osaka University Hospital (Approval Number: 885 [Osaka University Critical Care Consortium Novel Omix Project; Occonomix Project]). Informed consent for blood sample collection was obtained from all patients or their relatives and healthy volunteers. If the patient’s relatives withdrew consent at a later date or if patients regained the capacity to consent and requested to withdraw, the sample was discarded.

Whole-blood and single-cell RNA-seq analyses were performed. COVID-19 patients with ARDS admitted to the intensive care unit (ICU) of Osaka University Hospital between July 2020 and February 2021 were included in whole-blood RNA-seq analysis. COVID-19 patients with ARDS admitted to the ICU of Osaka University Hospital between May 2021 and October 2021 were included in single-cell RNA-seq analysis. The hospital accepts patients who have been transferred from other hospitals and have become severely ill, necessitating ventilator support. Healthy individuals who registered through general poster advertisements were considered controls. ARDS was diagnosed based on the Berlin definition (14). COVID-19 was diagnosed based on a positive result in the SARS-CoV-2 reverse transcription-polymerase chain reaction test and confirmed by pneumonia on chest computed tomography. Weaning from mechanical ventilation was performed according to a previously described protocol. Successful withdrawal from the ventilator was determined to have occurred when the following criteria were met: respiratory rate <35 breaths/min, SpO_2_ >90% after withdrawal, heart rate <140 bpm, systolic blood pressure 90–180 mmHg, and no symptoms of respiratory urgency (15). Cases requiring re-intubation within 24 h of initial intubation were also excluded.

### 2.2 Clinical data

The clinical data collected by the principal investigator from patients’ electronic medical records included age, sex, body mass index (BMI), Acute Physiology and Chronic Health Evaluation (APACHE) II score, Sequential Organ Failure Assessment (SOFA) score, comorbidities (hypertension, diabetes, and hyperlipidemia), mechanical ventilation duration, vaccination status, treatment details before and after specimen collection, and hospital outcomes.

### 2.3 Timing of measurement

Samples were collected on the first or second day of hospitalization (within 24 h of arrival). As the patients with COVID-19 were admitted to the ICU at the time of critical illness, all samples were collected when they became severely ill, necessitating ventilator support. The primary outcome was set to 28-day ventilator-free days (VFD_28_) to determine whether ARDS was alleviated and whether early weaning from mechanical ventilation was possible. Among the COVID-19 patients with ARDS, those with VFD_28_ not equal to zero were included in the ventilator weaning (VW) group, and those with VFD_28_ equal to zero were included in the non-ventilator weaning (NVW) group. Healthy individuals were defined as healthy controls.

### 2.4 Bulk RNA-seq of whole blood

#### 2.4.1 RNA isolation and library construction

This procedure was performed as reported previously (16). Total RNA was isolated from whole blood using the PAXgene™ blood RNA system (BD Biosciences, San Jose, CA, USA). After blood collection, the tubes were stored at −30 °C until further analyses. RNA was extracted using the PAXgene blood RNA kit (Qiagen Inc., Venlo, The Netherlands) according to the manufacturer’s protocol. Eluted RNA was dissolved in RNase-free water. RNA quality and quantity were evaluated using the Bioanalyzer 2100 system (Agilent Technologies, Santa Clara, CA, USA). RNA was converted into double-stranded cDNA libraries using the SMART-seq HT kit (Takara Bio Inc., Shiga, Japan) according to the manufacturer’s protocol. The libraries were quantified using an Illumina library quantification kit (Kapa Biosystems Inc., Wilmington, MA, USA), and fragment size distribution was determined using a bioanalyzer. The number of samples required to detect candidate RNAs of prognostic relevance was estimated by power analysis. Assuming approximately 27,000 expressed genes, an absolute log_2_ fold change of 1.0, and a false discovery rate (FDR) of 0.1, at least nine samples were needed to achieve 80% statistical power (**Supplementary Figure 1**).

#### 2.4.2 RNA-Seq and bioinformatics analysis

High-throughput sequencing was performed using an MGIseq 2000 system (MGI) with 100-bp paired-end reads, and the sequence reads were converted into FASTQ files. The FASTQ sequences were aligned using TopHat2 (17). The hg19 reference genome and corresponding index files provided by the TopHat website were used. The BAM files generated using Tophat2 were converted into raw count files using featureCounts (18). The raw count data was first organized into a DGEList object using the edgeR package (19). Lowly expressed genes were filtered out using the filterByExpr function. Library sizes were then normalized using the Trimmed Mean of M-values (TMM) method with the calcNormFactors function. To identify differentially expressed genes (DEGs) between the VW and NVW groups, we employed a quasi-likelihood negative binomial generalized linear model (GLM) approach within edgeR. The model was designed to account for potential confounding variables by including age and sex as covariates (~ group + age + sex). Dispersions were estimated using the estimateDisp function, and the GLM was fitted to the data with glmQLFit. Finally, the glmQLFTest function was used to perform a quasi-likelihood F-test to identify DEGs. Genes with a false discovery rate (FDR) less than 0.05 were considered statistically significant. For visualization and clustering, normalized expression values were calculated as log-transformed counts per million (logCPM). Hierarchical clustering of samples was performed based on Spearman's correlation distance and Ward's hierarchical clustering method ("ward.D"). A heatmap of the top 50 DEGs was generated using the pheatmap package (https://cran.r-project.org/web/packages/pheatmap/index.html), with expression values scaled per gene (Z-score) to visualize relative expression patterns.

### 2.5 Single-cell RNA-seq of neutrophils

#### 2.5.1 Neutrophil isolation and library construction

Whole blood, collected from patients or healthy controls, was first separated into serum, peripheral blood mononuclear cells, and a layer of red blood cells and neutrophils using gradient centrifugation with Histopaque (Sigma Aldrich). The lower layer of neutrophils and red blood cells was removed, and only the red blood cells were lysed using RBC buffer. The samples were washed several times using phosphate-buffered saline containing 2% bovine serum albumin. For neutrophil samples from healthy controls, TotalSeq-C hash tag antibody (BioLegend) staining was performed, as described previously (21). The cell number and viability of each sample were determined using a Countess II FL automated cell counter following trypan blue staining; the samples were then processed immediately for use using a 10X Genomics single-cell system, followed by the Chromium Next GEM single-cell V(D)J reagent kit v2 with feature barcoding technology for cell surface protein-rev D protocol. Gene expression and feature barcode libraries were prepared according to the manufacturer’s protocol (10x Genomics). All libraries were sequenced using DNBSEQ-G400 (MGI) to obtain at least 20,000 reads per cell for gene expression and 5,000 reads per cell for cell surface proteins.

#### 2.5.2 Bioinformatics analysis

Sequencing data obtained from MGISEQ-G400 were aligned to the GRCh38 genome using Cell Ranger (v.6.1.0) with default settings, including the “–force-cells 10000” option. The filtered matrices generated by Cellranger were loaded into the R package Seurat (v.4.0) (22), and data filtering, normalization, scaling, dimensional reduction, clustering, and visualization were performed using Seurat. For pseudo-bulk RNA-seq analysis, raw counts from each sample were aggregated. Differentially expressed genes (DEGs) and hierarchical clustering were analyzed using the edgeR package. A generalized linear model (GLM) was constructed to account for potential confounding factors by including age and sex as covariates (~ group + age + sex). After filtering lowly expressed genes, libraries were normalized using the TMM method. DEGs were identified using a quasi-likelihood F-test (glmQLFit and glmQLFTest). Hierarchical clustering of samples was performed using the distance calculated from Spearman's correlation with the "ward.D" method. Neutrophil classification into pro-neutrophils, pre-neutrophils, and mature neutrophils was performed according to the method reported by Schulte–Schrepping et al. (23), using established marker genes (ELANE, MPO, PRTN3, FUT4 for pro-neutrophils; PADI4, LCN2, ITGAM, CD101 for pre-neutrophils; and MME, FCGR3A for mature neutrophils). The proportions of pro-neutrophils, pre-neutrophils, and mature neutrophils were calculated for each patient. In addition, a UMAP was constructed for each patient.

To identify DEGs between VW and NVW, the FindMarkers function in Seurat was used, employing the MAST test. This model accounted for patient-to-patient variability by including the sample ID as a latent variable. This analysis was performed on all neutrophils combined, as well as within each neutrophil subset (pro-neutrophils, pre-neutrophils, and mature neutrophils) separately. Genes with an adjusted p-value < 0.1 were considered significantly differentially expressed. Gene Ontology (GO) enrichment analysis was subsequently performed on the identified DEGs using the enrichGO function in the clusterProfiler R package (20).

### 2.6 Statistical analysis

Summary data are presented as medians (interquartile ranges) for continuous variables and numbers (%) for categorical variables. Differences between the groups were detected using the Mann–Whitney *U* test for continuous variables and the chi-square test or Fisher’s exact test for nominal variables. Statistical analyses were performed using commercially available statistical analysis software (JMP Pro 16 software; SAS Institute Inc., Cary, NC, USA) (RRID: SCR_022199). P-values<0.05 were considered statistically significant.

## 3 Results

### 3.1 Patient characteristics

In whole-blood RNA-seq analysis, 28 COVID-19 patients with ARDS (9 in the NVW group and 19 in the VW group) and 16 healthy controls were included. The median ages of patients in the NVW and VW groups and healthy controls were 76, 72, and 55 years, respectively. Viral subtypes were identified in 19 of the cases, and all of which were of the 20B subtype. All patients with COVID-19 and ARDS received treatment in the ICU and were placed on mechanical ventilation. All cases included in this study were not vaccinated, and did not require re-intubation within 24 h of extubation. The median 28-day ventilator-free duration of the VW group was 15 days, and the mortality rate of the NVW group was 33.3% (**Table 1**). In the NVW group, except for fatal cases, all patients who could not be weaned from the ventilator remained dependent because of decreased oxygenation. There were no cases in which nonpulmonary complications, such as hypoxic encephalopathy, posed challenges for ventilator weaning. Clinical information is presented in **Supplementary Table 1**.

**Table 1:** Clinical findings of patients upon admission.

Variables	Patients with COVID-19 ARDS (*n* = 28)			Healthy controls (*n* = 16)
	Non-ventilator weaning group (*n* = 9)	Ventilator weaning group (*n* = 19)	p-value	
Age (IQR), years	76 (64–84)	72 (62–77)	0.28	55 (34–59.5)
Male sex, No. (%)	6 (66.7)	12 (63.2)	1.00	8 (50)
BMI (IQR), kg/m^2^	22.2 (20.1–23.9)	23.4 (22.6–28.4)	0.07	22.0 (20.3–24.6)
White blood cell (IQR), ×10^3^/μL	8.23 (6.08–15.94)	8.52 (4.78–9.82)	0.64	
Neutrophils (IQR), % ^a^	89.6 (87–93.2)	88.8 (84.8–94.4)	0.57	
Lymphocytes (IQR), % ^a^	7.3 (4.05–10.5)	7.3 (4.4–11.7)	0.61	
Neutrophil-to-lymphocyte ratio (IQR)	12.12 (8.45–23.01)	12.16 (7.35–21.45)	0.62	
APACHE II score (IQR)	20 (13–23)	13 (10–18)	0.04	–
SOFA score (IQR)	6 (5–8.5)	6 (3–7)	0.18	–
Comorbidity, No. (%)				
Hypertension	3 (33.3)	11 (57.9)	0.42	2 (12.5)
Diabetes	5 (55.6)	9 (47.4)	1.00	1 (6.3)
Kidney disease	3 (33.3)	3 (15.8)	0.35	0 (0)
Therapeutic intervention prior to sample collection, No. (%)				
Vaccination	0 (0)	0 (0)	1.00	–
Steroid infusion	6 (66.6)	13 (68.4)	0.93	–
Ciclesonide	3 (33.3)	8 (42.1)	0.66	–
Tocilizumab	1 (11.1)	1 (5.26)	0.59	–
Remdesivir	0 (0)	6 (31.6)	0.02	–
Therapeutic intervention after sample collection, No. (%)				
Steroid infusion	9 (100)	19 (100)	1.00	–
Ciclesonide	0 (0)	0 (0)	1.00	–
Tocilizumab	0 (0)	0 (0)	1.00	–
Remdesivir	2 (22.2)	9 (47.4)	0.19	–
28 ventilator-free days (IQR)	0 (0-0)	15 (10–21)	<0.001	–
Hospital death, No. (%)	3 (33.3)	0 (0)	0.03	–

a Ratio of neutrophil and lymphocyte counts to white blood cell count. Blood sampling results on admission. Abbreviations: COVID-19, coronavirus disease 2019; ARDS, acute respiratory distress syndrome; IQR, interquartile range; BMI, body mass index; APACHE, Acute Physiology and Chronic Health Evaluation; SOFA, Sequential Organ Failure Assessment

In the single-cell analysis cohort, we enrolled five COVID-19 patients with ARDS and six healthy volunteers. **Table 2** summarizes the clinical and demographic characteristics of the patients and healthy controls. Testing for viral subtypes was performed in two cases: 20B and 20I. One case was vaccinated. One patient died during hospitalization, and two required mechanical ventilation for >90 days. Similarly, the difficulty in weaning patients from mechanical ventilation within 28 days were attributed to decreased oxygenation. **Supplementary Table 2** provides details of the clinical outcomes of COVID-19 patients with ARDS.

**Table 2:** Clinical and demographic characteristics of patients and healthy controls in the single-cell analysis cohort.

Variables	Patients with COVID-19	Healthy controls	p-value
	(*n* = 5)	(*n* = 6)	
Male sex, No. (%)	5 (100)	2 (33.3)	0.06
Age, median years (IQR)	63 (69–71)	74 (48–76.8)	0.41
Age group, n (%)			0.21
20–34 years	0 (0)	0 (0)	
35–49 years	0 (0)	2 (33.3)	
50–64 years	2 (40)	0 (0)	
65–79 years	3 (60)	4 (66.7)	
>80 years	0 (0)	0 (0)	
Comorbidities, No. (%)			
Heart disease ^a^	0 (0)	0 (0)	1.0
Lung disease ^b^	0 (0)	0 (0)	1.0
Kidney disease ^c^	2 (40)	0 (0)	0.18
Immunocompromised condition ^d^	0 (0)	0 (0)	1.0
Hypertension	4 (80)	3 (50)	0.55
Diabetes	2 (40)	0 (0)	0.19
BMI, kg/m^2^, median (IQR)	26.2 (24.8–29.4)	21.5 (17.4–25.5)	0.1
BMI, No. (%)			0.24
0–24.9 kg/m^2^	2 (40)	5 (83.3)	
25.0–39.9 kg/m^2^	3 (60)	1 (16.7)	
>40 kg/m^2^	0 (0)	0 (0)	
Unknown	0 (0)	0 (0)	
Therapeutic intervention prior to sample collection, No. (%)			
Vaccination	1 (20)	–	
Steroid infusion	4 (80)	–	
Ciclesonide	0 (0)	–	
Tocilizumab	1 (20)	–	
Remdesivir	3 (60)	–	
Therapeutic intervention after sample collection, No. (%)			
Steroid infusion	5 (100)	–	
Ciclesonide	0 (0)	–	
Tocilizumab	0 (0)	–	
Remdesivir	0 (0)	–	

Data are reported as number (percentage) with mean ± standard deviation (SD) or median (IQR) as appropriate. P-values are for the comparisons between patients with COVID-19 and healthy volunteers.

a Heart disease includes coronary artery disease, congestive heart failure, and valvular disease.

b Lung disease includes asthma, chronic obstructive pulmonary disease, requiring home O2, and any chronic lung condition.

c Kidney disease includes chronic kidney disease, baseline creatinine >1.5.

d Immunocompromised conditions include active cancer, chemotherapy, transplant and use of immunosuppressant agents, and an asplenic condition.

Abbreviations: BMI, body mass index; COVID-19, coronavirus disease 2019; IQR, interquartile range.

### 3.2 Bulk RNA-seq of whole blood

**Figure 1:**
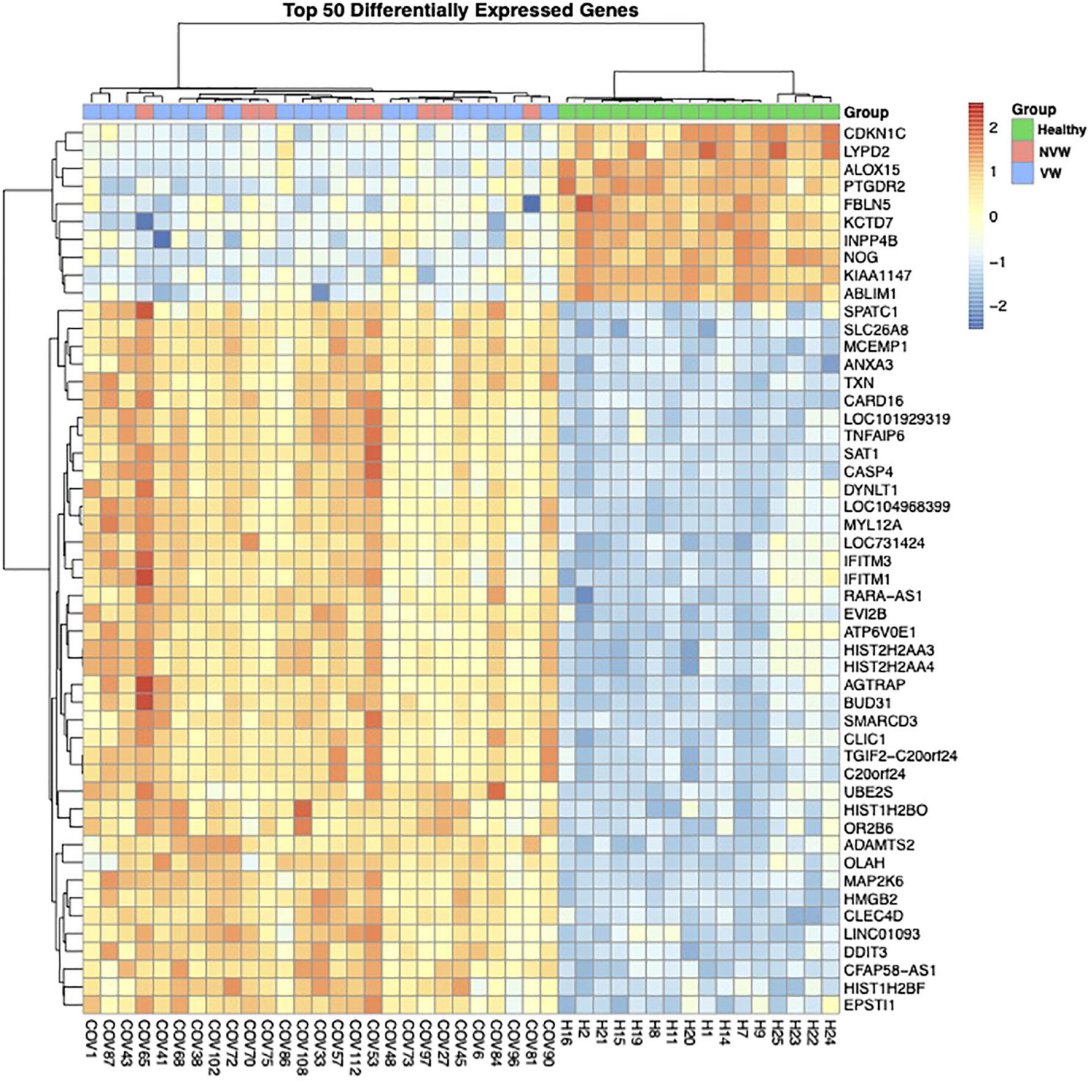
Dendrogram and heatmap based on normalized count data obtained from whole-blood RNA sequencing data. The dendrogram was obtained through hierarchical clustering using Ward’s method.

To investigate the differences in gene expression between the NVW and VW groups, we performed RNA-seq analyses. Hierarchical clustering was performed based on normalized count data calculated using edge R (**Supplementary Table 3**, **Figure 1**). Although COVID-19 patients with ARDS and healthy controls were clearly grouped, differences between the NVW and VW groups were not apparent. Similarly, in the bootstrap analysis, both VW and NVW groups exhibited low bootstrap values, making it difficult to distinguish between them based on clinical course (**Supplementary Figure 2**).

Between the COVID-19 patients with ARDS and healthy controls, 4,693 genes showed significant changes in expression (FDR<0.1), with upregulated expression of several genes related to viral gene expression in healthy controls and of genes related to neutrophil activation in the COVID-19 patients with ARDS (**Supplementary Figure 3**). In the NVW and VW groups, only one gene encoding the folate receptor gamma showed changes in expression (**Supplementary Table 4**). Clear differences were not observed between the NVW and VW groups with respect to bulk RNA-seq of whole blood. Despite no significant differences in gene expression between the NVW and VW groups, the GO enrichment analysis performed on genes with upregulated expression in the NVW group showed that genes related to neutrophil activation were enriched (**Supplementary Figure 4**). Therefore, we decided to focus on neutrophils rather than whole blood.

### 3.3 Single-cell RNA-seq of neutrophils

Neutrophils were isolated from blood cells, and single-cell RNA-seq analysis was performed using the 10X Genomics Chromium system. Cells with low expression levels and non-neutrophil cells were removed, following which 35,759 cells were obtained. Hierarchical clustering was first performed using the normalized gene expression levels of all cells (pseudo-bulk RNA-seq).

**Figure 2:**
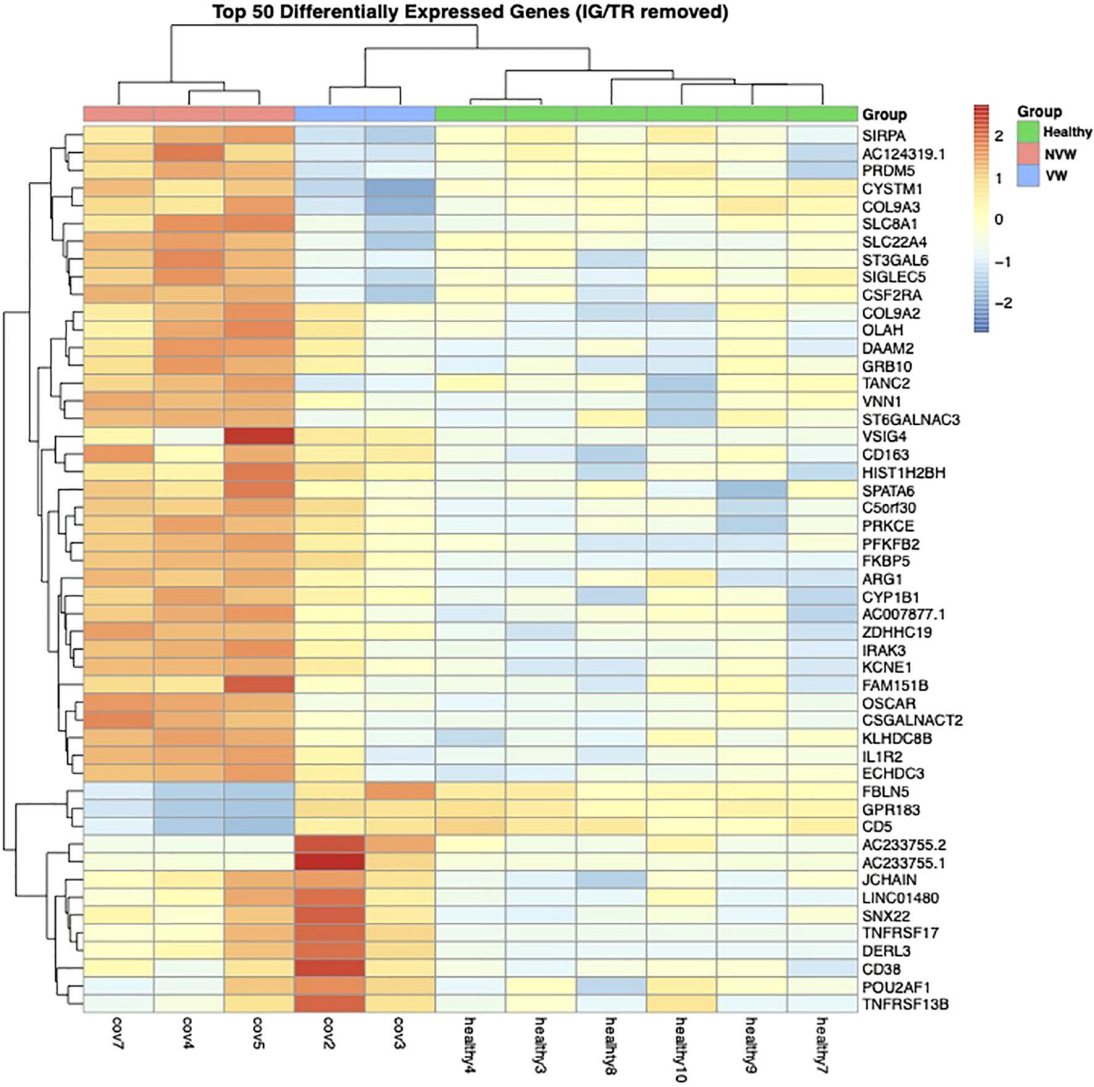
Dendrogram and heatmap based on normalized count data obtained from neutrophil single-cell RNA sequencing data. The dendrogram was obtained through hierarchical clustering using Ward’s method.

The pseudo-bulk RNA-seq analysis revealed clear differences between the healthy controls and COVID-19 patients with ARDS and between the VW and NVW groups (**Figure 2**). Patient cases 1 and 2 were classified into one cluster, six healthy individuals into another cluster, and patient cases 3, 4, and 5 into a separate cluster. The cluster of patient cases 1 and 2 corresponded to the VW group, whereas those of cases 3, 4, and 5 corresponded to the NVW group, resulting in the same classification as the cluster classified by clinical course; this indicated that it was possible to wean patients off mechanical ventilation within 28 days. Bootstrap analysis showed that it was possible to distinguish between the VW and NVW groups (**Supplementary Figure 5**).

**Figure 3:**
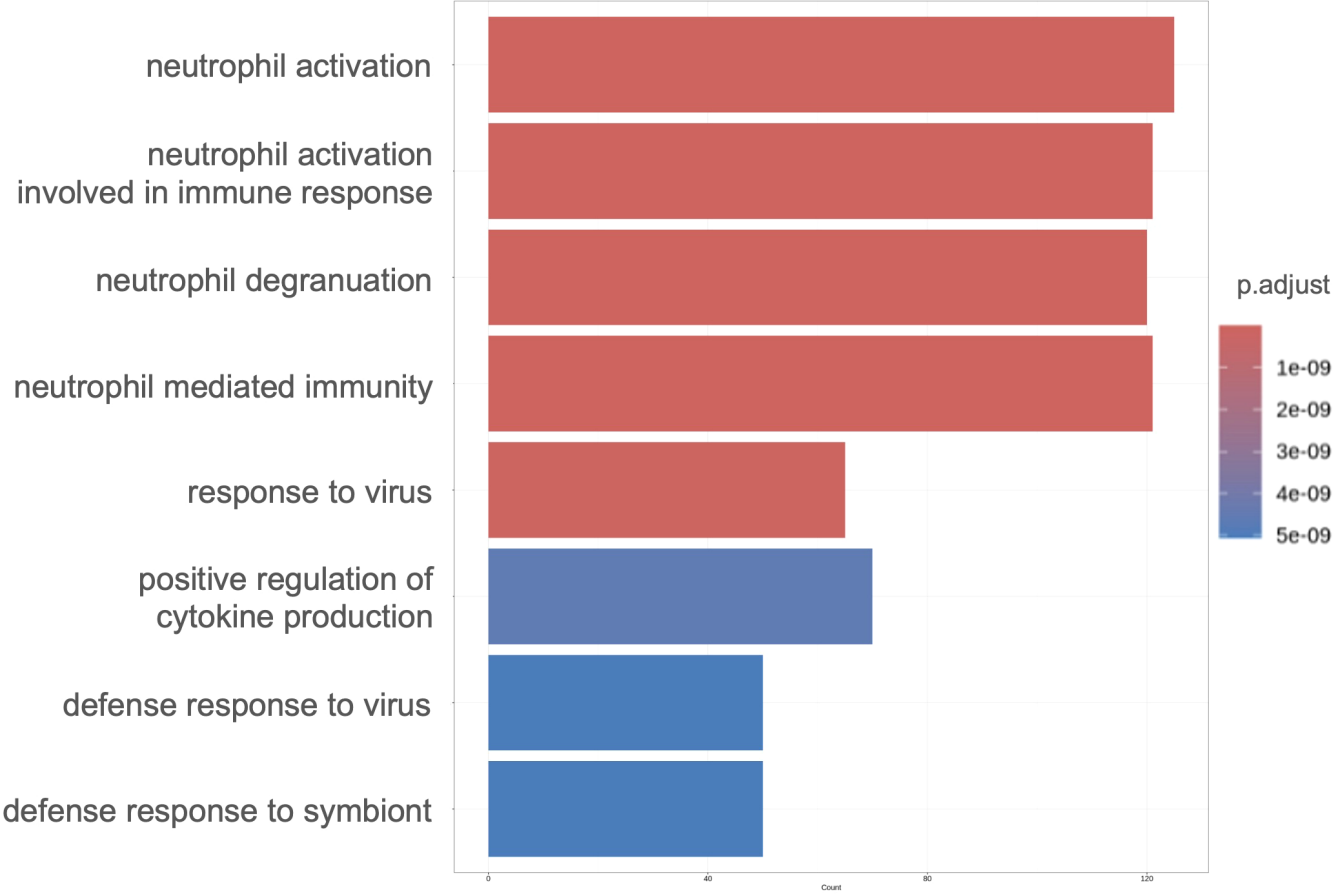
Gene ontology enrichment analysis of significantly upregulated genes in the NVW group. The top eight enriched ontologies are shown. VW, ventilator weaning; NVW, non-ventilator weaning.

**Figure 4:**
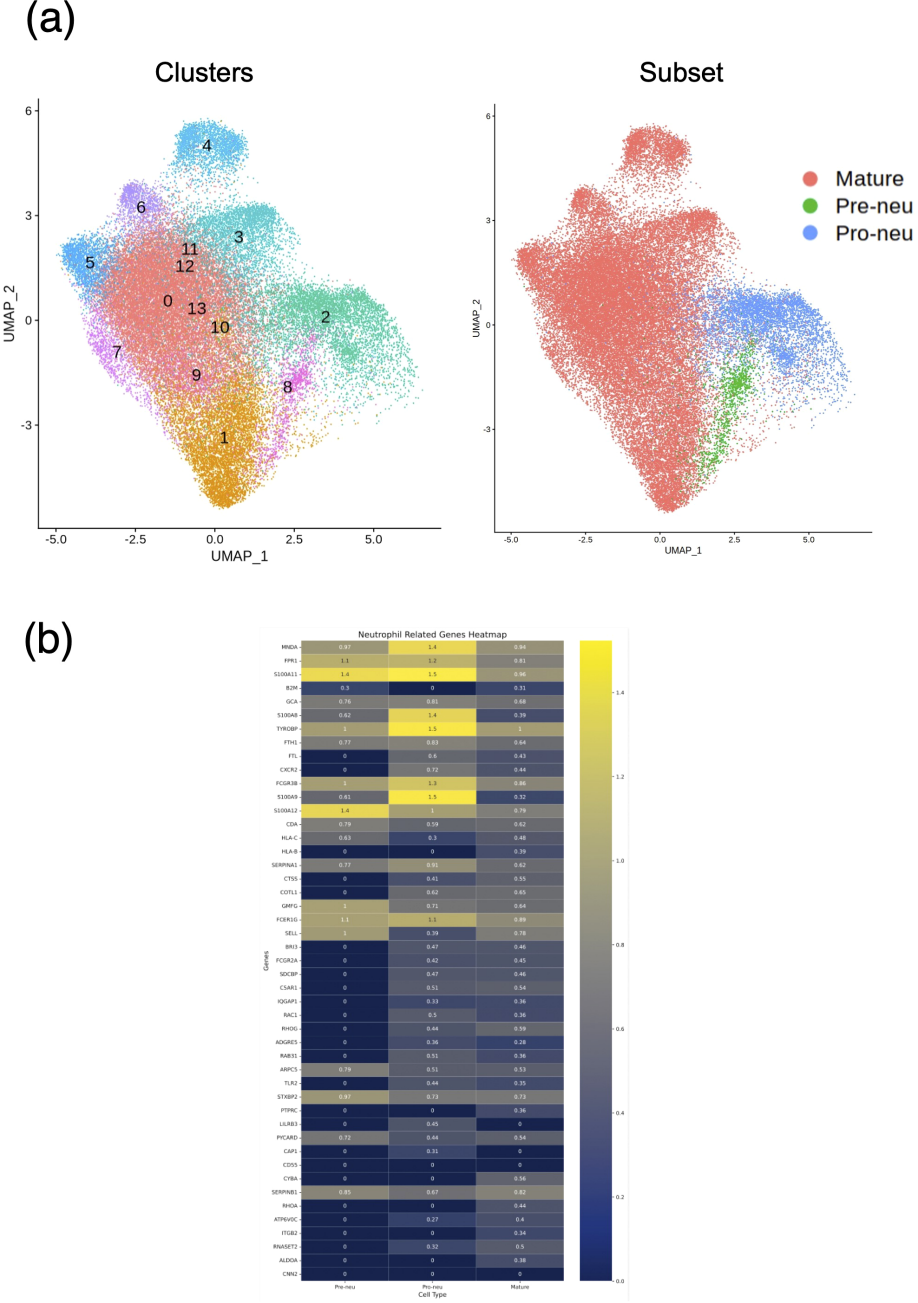
Single-cell RNA sequencing of neutrophils. **(a)** UMAP projection of neutrophils colored by Seurat clusters (left) and subsets (right). **(b)** Heat map displaying neutrophil-related genes with high expression in the NVW group. VW, ventilator weaning; NVW, non-ventilator weaning.

Our results suggested a relationship between gene expression and neutrophil maturity at the time of severe illness and clinical course, indicating the possibility of weaning off mechanical ventilation. Therefore, we performed differential expression analysis of all neutrophil-related genes between the VW and NVW groups. We identified 2,596 genes with significant changes (p_val_adj<0.1), of which 1,095 genes were upregulated and 1,501 genes were downregulated in the NVW group (**Supplementary Table 5**). Many genes related to neutrophil activation were found among the significantly upregulated genes in the NVW group (**Figure 3**; **Supplementary Table 6**). To identify neutrophils showing the most variable expression, neutrophils were clustered using single-cell RNA-seq data. This resulted in 14 distinct clusters, which were then annotated according to the method reported by Schulte-Schrepping et al. (23) (**Figure 4A**). The validity of the neutrophil cell counts used in the single-cell RNA-seq analysis (24) was retrospectively assessed. The cell count required to classify neutrophils into three subpopulations, specifically, mature neutrophils, pre-neutrophils, and pro-neutrophils, was estimated to be 290 cells. In this analysis, the cell counts used were 30216 cells for mature neutrophils, 772 cells for pre-neutrophils, and 4771 cells for pro-neutrophils. These counts were deemed sufficient to accurately classify neutrophils into the three subpopulations (**Supplementary Figure 6**). Among the three subsets, genes related to neutrophil activation were highly differentially expressed in the pro-neutrophil subset compared with those in the other subsets (**Figure 4B**). *S100A8, FCGR3B, MNDA, S100A11, FPR1, TYROBP*, and *S100A9* were the most differentially expressed (log_2_ fold>1.2) in pro-neutrophils. In the subsets of each sample, the NVW group exhibited a subset composition similar to that of healthy individuals, whereas the VW group differed from healthy individuals, showing an increase in pro-neutrophils (**Supplementary Figure 7**, **Supplementary Figure 8**; **Supplementary Table 7**). In **Figure 4B**, particularly in the pro-neutrophil genes, the NVW group showed greater changes than the VW group. Single-cell analysis enabled the assessment of gene expression levels within each subset.

## 4 Discussion

In this study, we analyzed samples from COVID-19 patients at the onset of critical illness requiring mechanical ventilation. Single-cell analysis of neutrophils enabled the identification of clinical features that may not be evident through whole blood analysis. These exploratory findings suggest a potential association between neutrophil-specific genetic information and clinical parameters.

Despite a difference in the APACHE II score of patients in the VW and NVW groups at the time of admission, the SOFA score, neutrophil-to-lymphocyte ratio, or neutrophil proportion did not differ (**Supplementary Figure 9**), which has been reported to be associated with in-hospital mortality (25) and the need for mechanical ventilation (26). The expression of genes related to neutrophil activation is higher in COVID-19 patients than in healthy controls (12). Neutrophil activation is higher in severe cases (WHO ordinal scale: 5–7) than in mild cases (WHO ordinal scale: 1–4) (13). We also compared our analysis with the single-cell RNA data analyzed by Trump et al. (27), specifically focusing on the examination of neutrophil gene expression between the critical and severe cohorts. The results were consistent with those of the present study, showing higher expression of genes involved in neutrophil activation in critical cohorts compared to the severe cohorts (**Supplementary Figure 10**). These results indicate a potential link between our single-cell RNA-seq data and patients’ clinical information.

However, few studies have evaluated the association between clinical outcomes, such as weaning from mechanical ventilation within 28 days after infection, and neutrophil profiles using both bulk RNA-seq and single-cell analysis in similar disease settings. In the present study, bulk RNA-seq did not reveal any clear differences in the expression of genes related to neutrophil activation between the VW and NVW groups (**Figure 1**). However, gene expression in neutrophils differed between the two groups (**Figure 2**). As the gene expression level in neutrophils is lower than that in lymphocytes, it cannot be detected using whole-blood RNA-seq. Moreover, a difference in neutrophil-to-lymphocyte ratio between the VW and NVW groups was observed on day 7 of admission (**Supplementary Figure 11**), although there was no difference at the time of admission (**Supplementary Figure 9**). Single-cell analysis of neutrophils might provide complementary information to the neutrophil-to-lymphocyte ratio, which has been linked to clinical information. It is also noteworthy that single-cell analysis of neutrophils enabled the evaluation of clinical features that could not be assessed through whole blood analysis.

The ability to predict ventilator withdrawal may assist clinicians in making informed decisions regarding medication management and ventilator discontinuation, potentially improving patient outcomes (28). Although ventilator parameters have been used to predict ventilator discontinuation previously (29), there have been no previous reports of such evaluations being carried out based on gene expression. Our single-cell sequencing analysis results showed that the expression of neutrophil activation-related genes was significantly upregulated in the NVW group compared to the VW group (**Figure 3**) and that the expression of these genes was upregulated in the pro-neutrophil subset (**Figure 4**). A strong association has been identified between neutrophils in the blood of COVID-19 patients and their clinical condition (25, 30, 31), and various reports have described the presence of pro-neutrophils and pre-neutrophils in the blood of critically ill patients (22, 32, 33).

Pro-neutrophils express genes that are involved in NET formation (22, 34). As NETs play an important role in COVID-19 severity (35), pro-neutrophil activation in this study may be related to COVID-19 severity. *S100A8* was one of the most upregulated gene in the NVW group of pro-neutrophils (**Figure 4B**). S100A8 is a calcium- and zinc-binding protein that plays an important role in regulating inflammatory processes and immune responses (36). Inflammation triggers the release of S100A8, stimulating premature neutrophil mobilization on binding to Toll-like receptor 4 (37, 38). S100A8 forms a heterodimer with S100A9 (39), which was also upregulated 2.8-fold in our study (**Figure 4B**). S100A8 induces a set of key cytokines/chemokines such as *CCL2*, vascular endothelial growth factor, CXCL1, and tumor necrosis factor, resulting in the recruitment of immune-suppressive myeloid cells, which induce NET formation (40); S100A8 may be a candidate marker of severe symptoms. In this study, the S100A8 level in pro-neutrophils increased by 2.6 times (**Figure 4B**). The patients in this study had severe prognoses, and our genomic approach could potentially contribute to the recognition of patients’ clinical information.

In the initial response to lung injury, innate immune cells circulating throughout the body are mobilized to the endothelium and epithelium of the injured alveoli, resulting in inflammation and tissue damage (7). Neutrophils are innate immune cells that play a critical role in immune response. ARDS is primarily a lung disease, and lung function parameters, such as the PaO_2_/FiO_2_ ratio, are commonly determined to assess ARDS pathogenesis. However, in our study, we analyzed circulating neutrophils rather than neutrophils in the lungs. As neutrophils in the peripheral blood are among the most abundant cells responsible for innate immunity and can be conveniently collected in clinical settings, the study results are of considerable clinical importance. All cases in which it was difficult to wean patients from the ventilator were attributable to hypoxemia, which may also reflect the pathophysiology of ARDS.

Our study has certain limitations. First, it was conducted at a single institution with a relatively small number of participants. Every effort was made to collect samples where possible, but in some cases, collection was difficult due to medical reasons. Although our department possesses the equipment to collect and process samples rapidly, general hospitals may find it challenging to avail this facility; therefore, our observations may be associated with a facility bias. We conducted a verification based on an analysis following the sample size calculation. However, owing to constraints in the analytical methodology, sample size was limited, and validation could not be performed. Accordingly, generalizing the observed association between single-cell analysis of peripheral neutrophils and ventilator weaning in this study is challenging. Nevertheless, the exploratory identification of gene expression profiles in neutrophils may hold future clinical relevance. To enhance generalizability, further analyses using a larger sample size are necessary.

Second, the median age of healthy controls was 55 years, which was lower than that of the COVID-19 patients with ARDS; therefore, age may have affected the study results. Further, we followed the protocol of a previous study for weaning patients from the ventilator (15). However, the treatment for each patient was individualized, which may have introduced selection bias. Lastly, patients with comorbidities such as hypertension and chronic renal failure were included in the analysis, and comorbidities may have influenced neutrophil gene expression. Our results may also be influenced by virus subtypes and vaccination, and the treatment received before the onset of the severe condition. However, single-cell analysis was performed only in one case in which vaccination was administered, making it difficult to determine any potential influence of the vaccination. Additionally, treatments with steroids and remdesivir may have affected the gene profiles. Nevertheless, a distinctive feature of this study is the evaluation of the neutrophil transcriptome during the onset of severe disease, along with clinical information, such as weaning from mechanical ventilation. Nevertheless, our findings may support the exploratory identification of genomic factors, such as neutrophil gene expression, that could be relevant to clinical parameters.

## Data Availability

The bulk and single-cell RNA-seq data used in this study have been deposited in NCBI’s Gene Expression Omnibus and are accessible through the accession numbers GSE199816 (https://www.ncbi.nlm.nih.gov/geo/query/acc.cgi?acc=GSE199816) and GSE179850 (https://www.ncbi.nlm.nih.gov/geo/query/acc.cgi?acc=GSE179850) for bulk RNA-seq and GSE234904 (https://www.ncbi.nlm.nih.gov/geo/query/acc.cgi?acc=GSE234904) for the single cell RNA-seq. The code used for the bioinformatic analyses in this study is available through GitHub at https://github.com/amufaamo/covid-neutrophil-ards/blob/main/scripts.md.
