# Supplementary material for: Neutrophil gene expression in COVID-19 patients with acute respiratory distress syndrome
Source: Front Immunol. 2025 Nov 6;16:1620745. doi: 10.3389/fimmu.2025.1620745 (PMC12631193; doi:10.3389/fimmu.2025.1620745)
Supplement: Supplementary file 1 [file Image1.pdf]

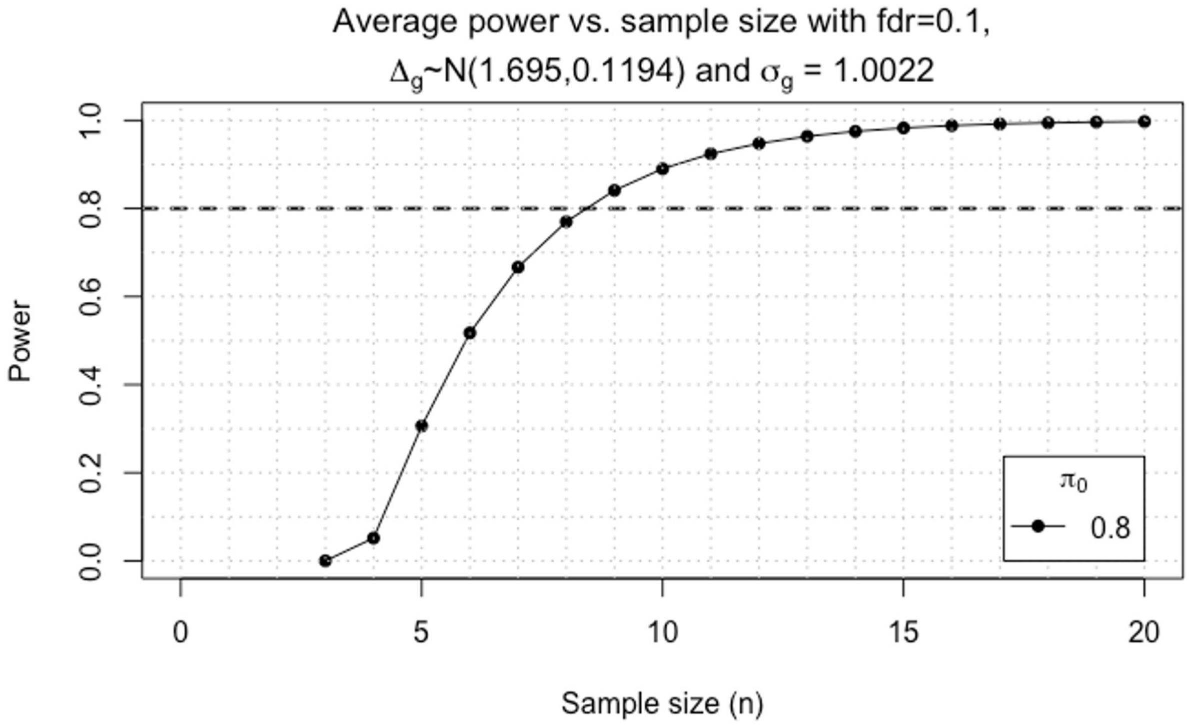

Supplementary Figure 1. Sample size analysis of Bulk RNA-seq
