# Supplementary figures and images for: Neutrophil gene expression in COVID-19 patients with acute respiratory distress syndrome
Source: Front Immunol. 2025 Nov 6;16:1620745. doi: 10.3389/fimmu.2025.1620745 (PMC12631193; doi:10.3389/fimmu.2025.1620745)

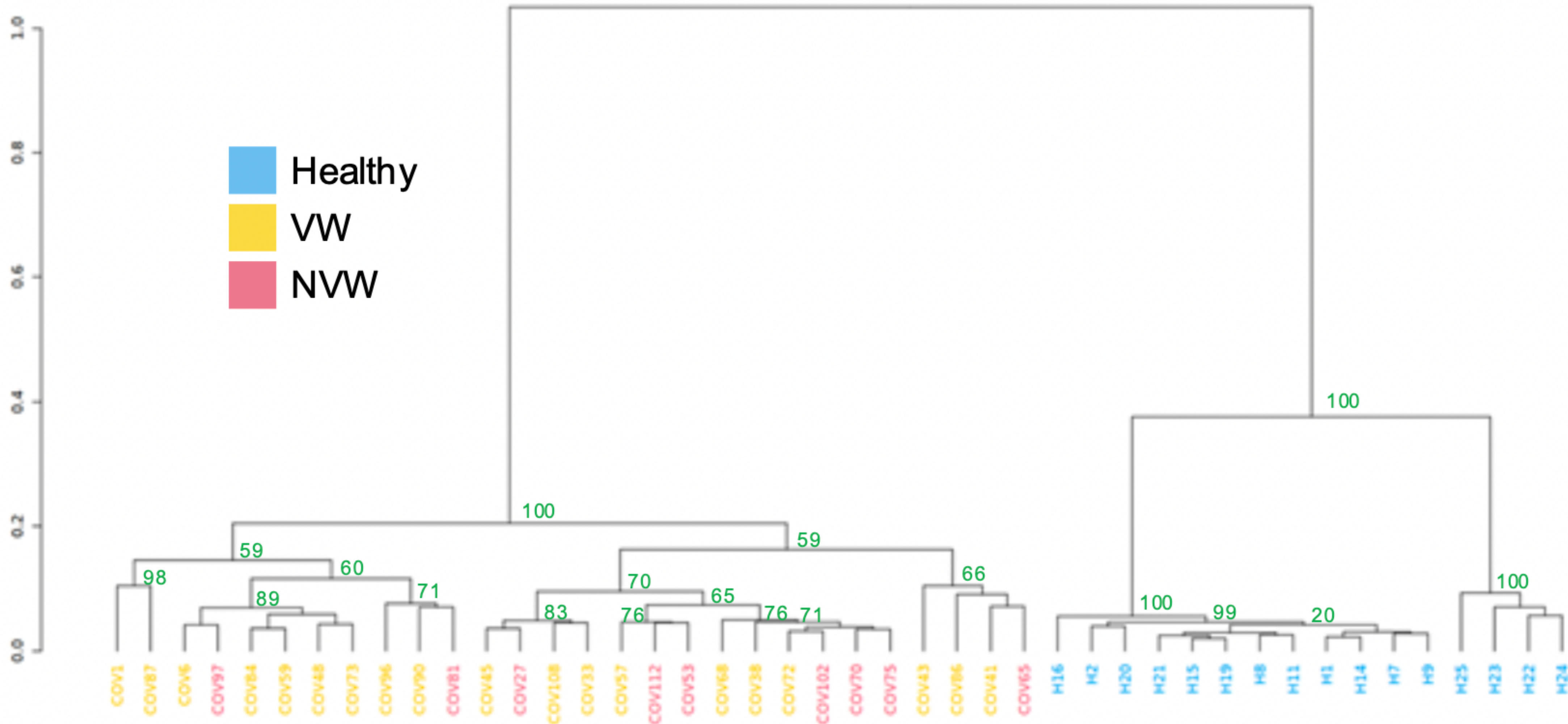

Supplement: Supplementary file 2 [file Image2.pdf]

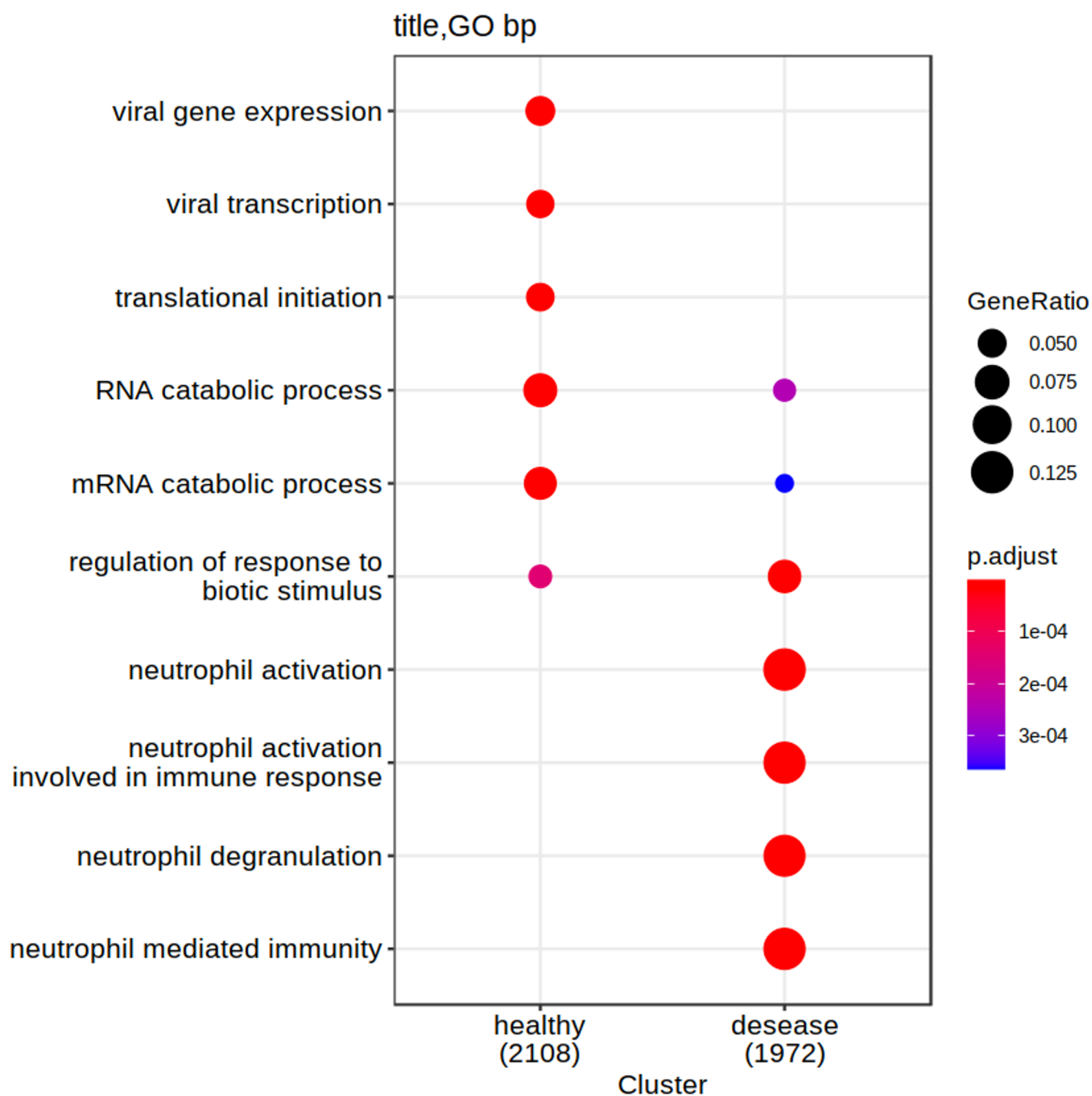

Supplementary Figure 3. GO enrichment bulk rna-seq healthy vs disease

Supplement: Supplementary file 3 [file Image3.pdf]

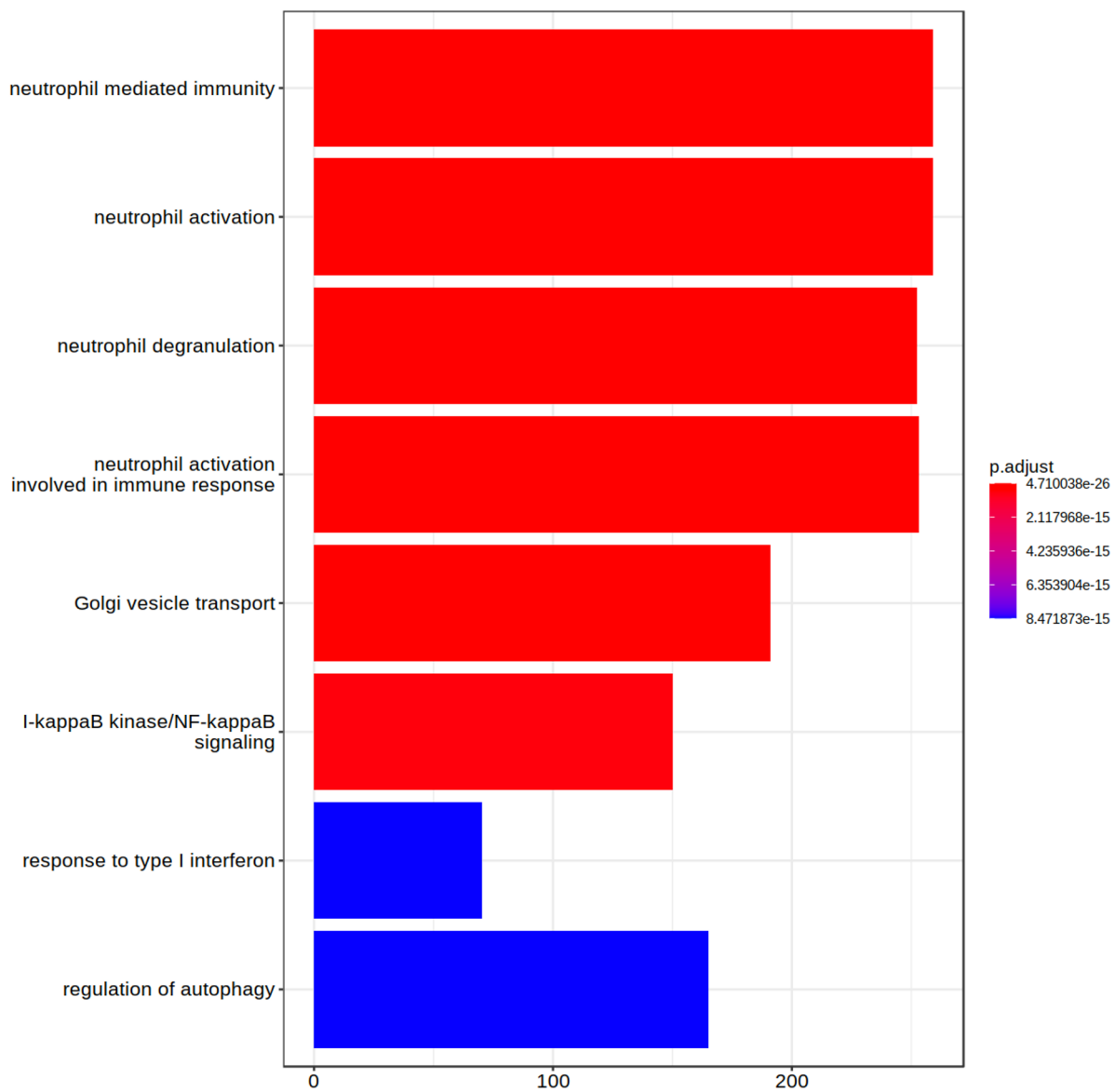

Supplementary Figure 4. GO term enriched with genes highly expressed in NVW.

Supplement: Supplementary file 4 [file Image4.pdf]

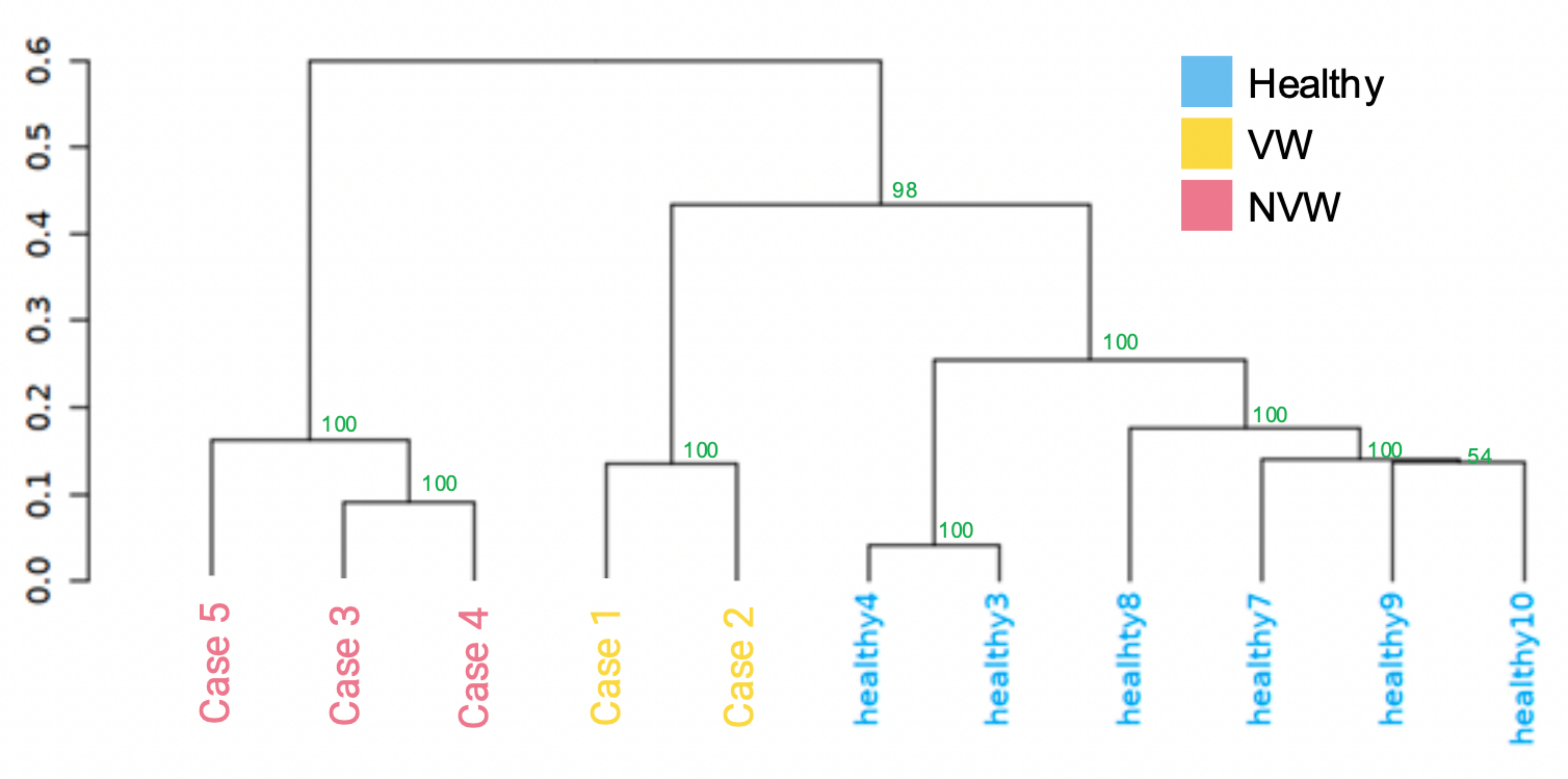

Supplement: Supplementary file 5 [file Image5.pdf]

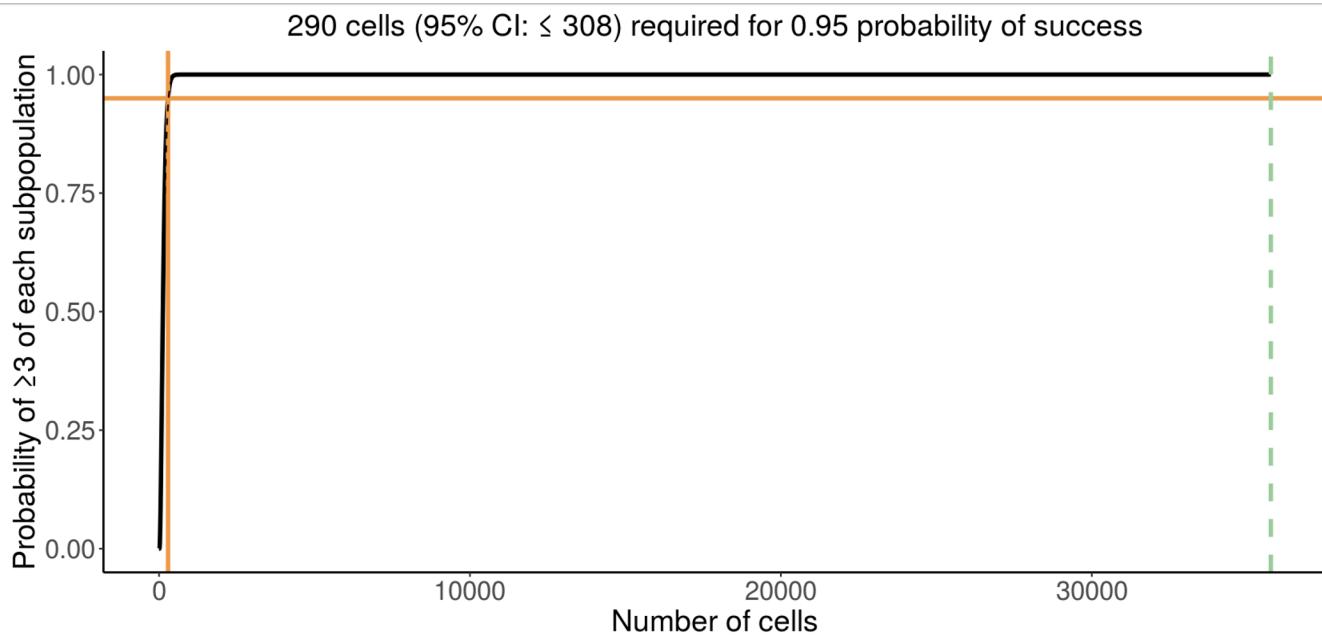

Supplementary Figure 6. Sample size analysis of single-cell RNA-seq

Supplement: Supplementary file 6 [file Image6.pdf]

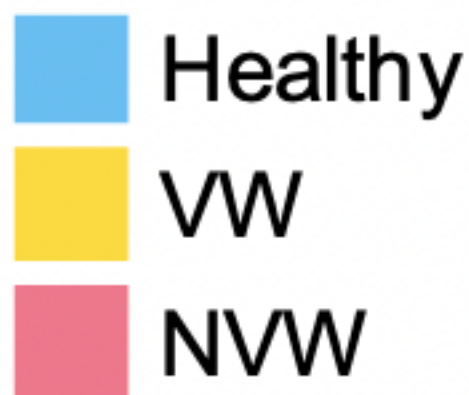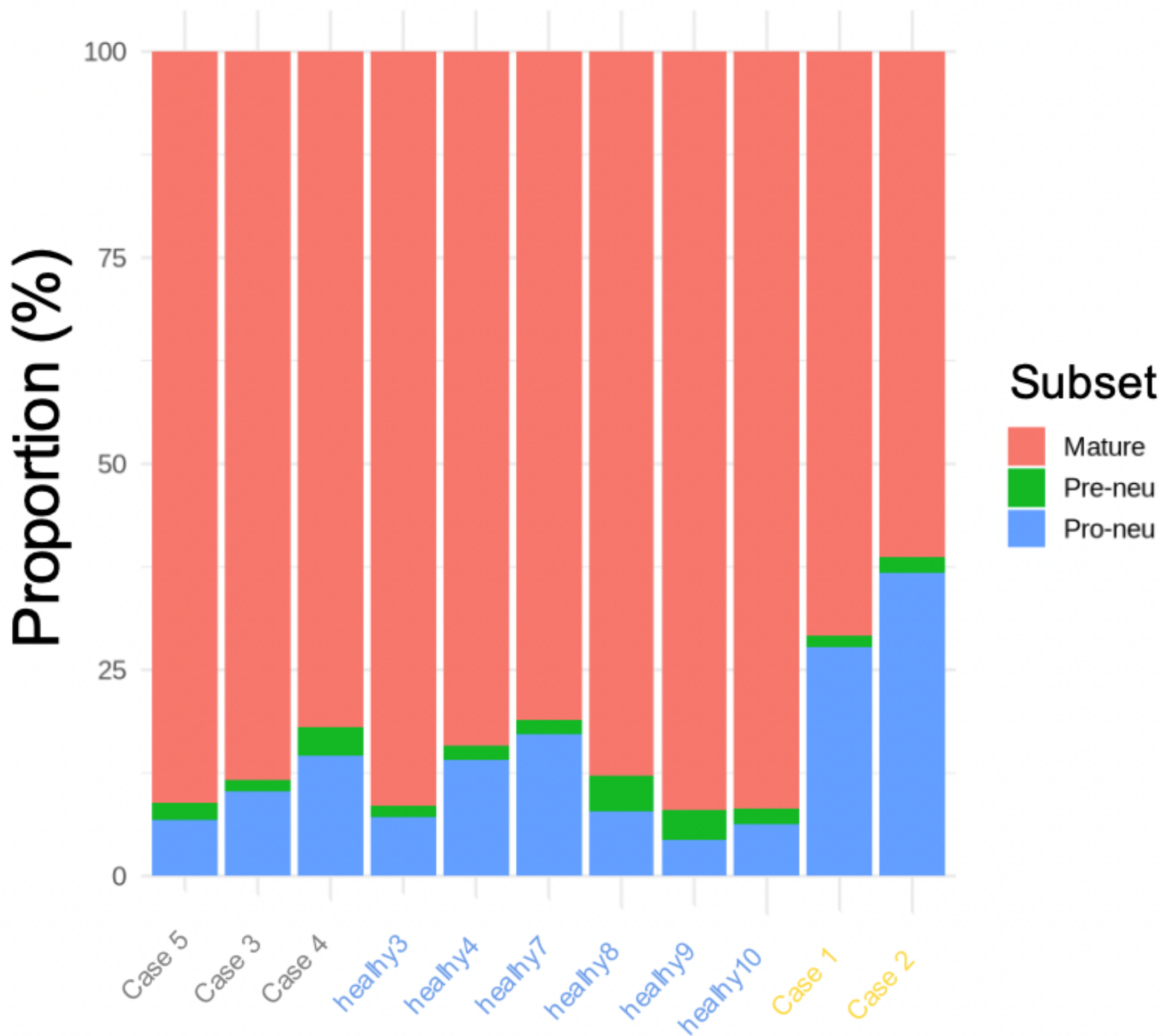

Supplement: Supplementary file 7 [file Image7.pdf]

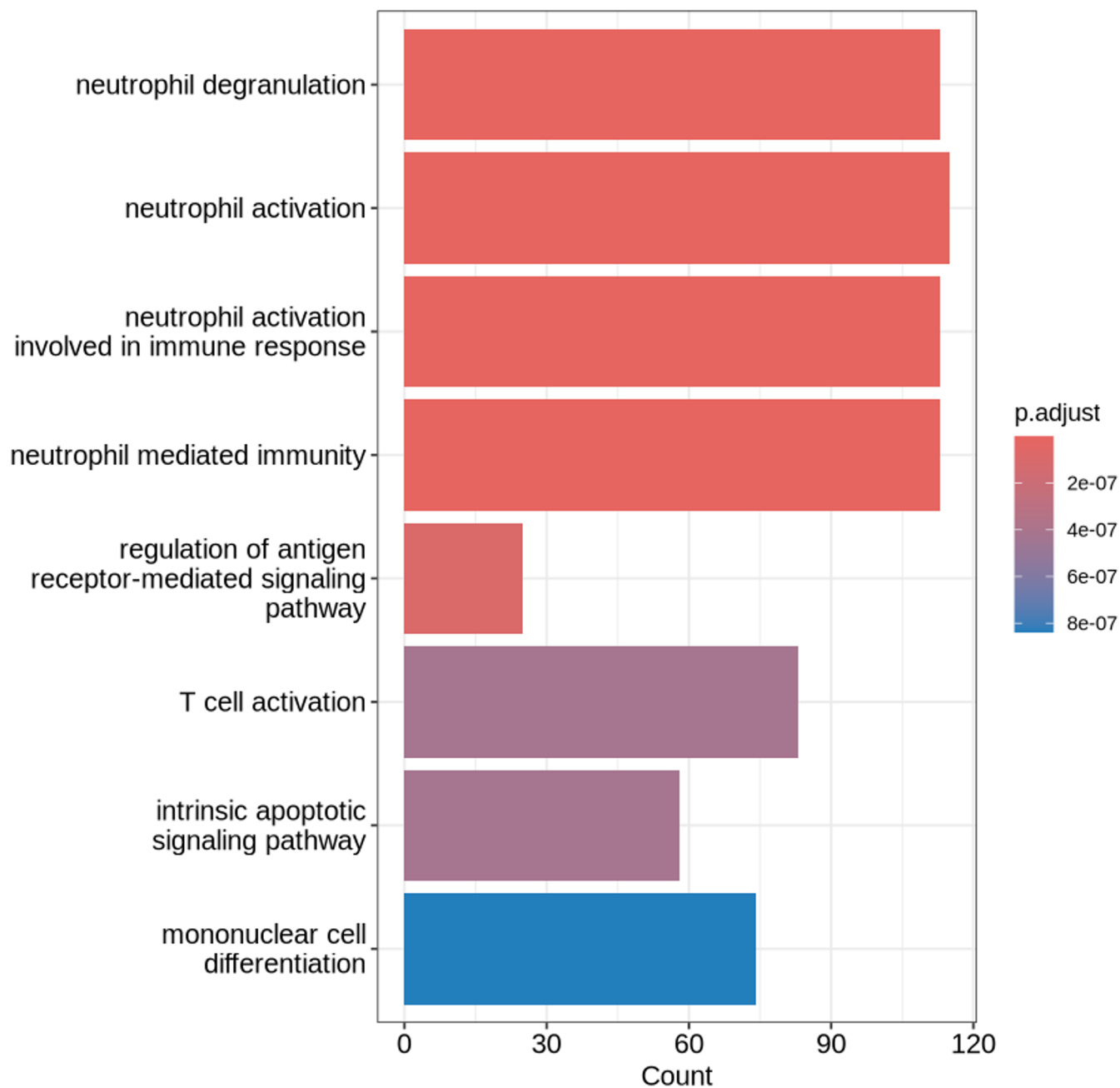

Supplementary Figure 10. GO terms which is enriched in critical cases compared to severe cases

Supplement: Supplementary file 10 [file Image10.pdf]

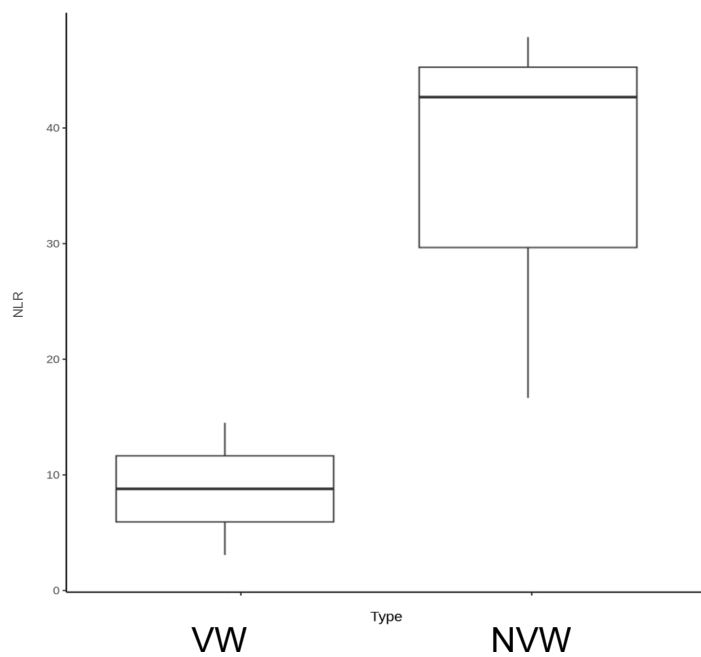

Supplementary Figure 11. Boxplot of neutrophil-to-lymphocyte ratio on day 7 of admission

Supplement: Supplementary file 11 [file Image11.pdf]
