# Supplementary material for: Neutrophil gene expression in COVID-19 patients with acute respiratory distress syndrome
Source: Front Immunol. 2025 Nov 6;16:1620745. doi: 10.3389/fimmu.2025.1620745 (PMC12631193; doi:10.3389/fimmu.2025.1620745)
Supplement: Supplementary file 8 [file Image8.pdf]

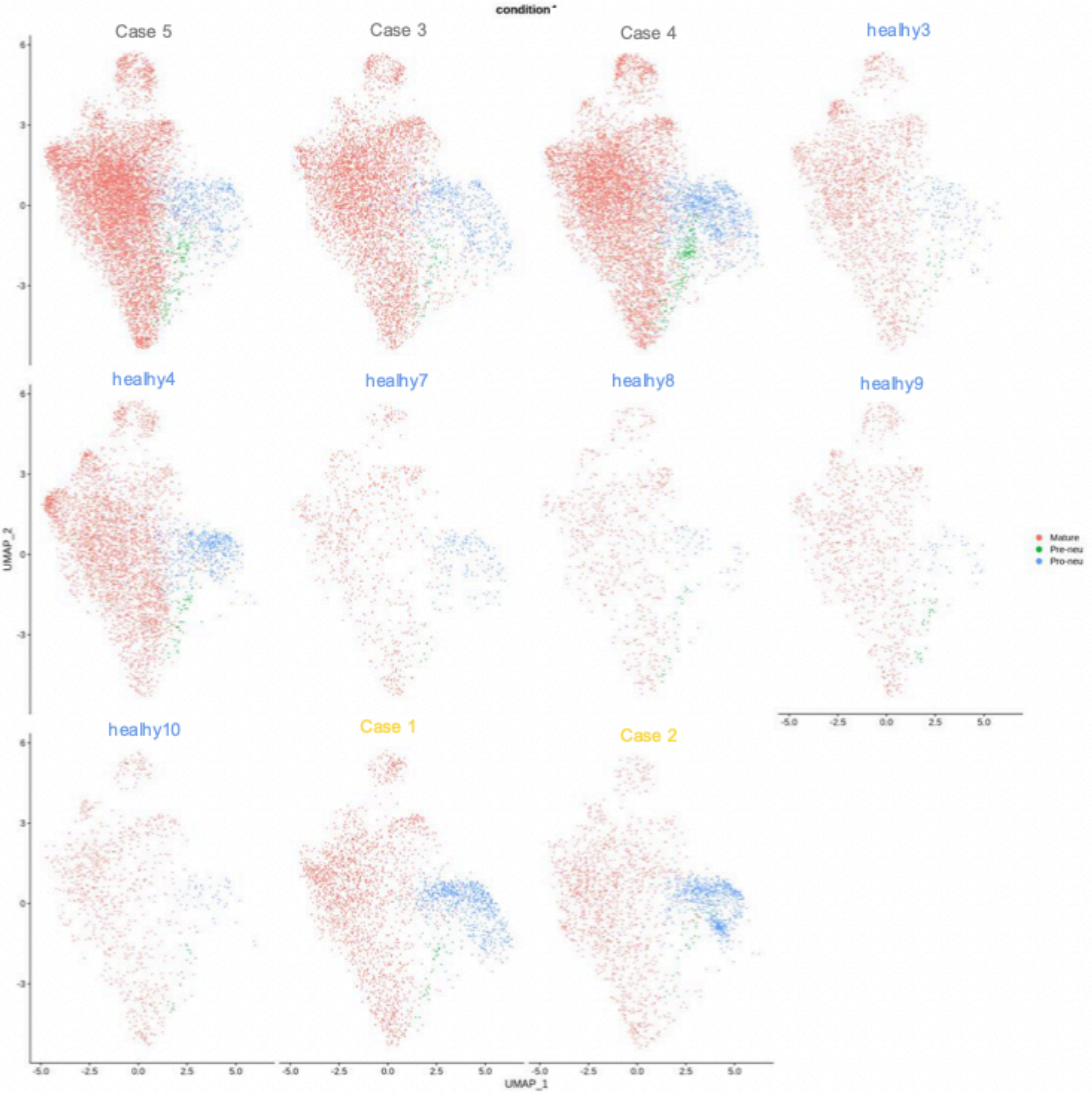
condition
Case 5
Case 3
Case 4
healhy3
healhy4
healhy7
healhy8
healhy9
healhy10
Case 1
Case 2
UMAP_2
UMAP_1
Mature
Pre-neu
Pro-neu
