# Supplementary material for: Neutrophil gene expression in COVID-19 patients with acute respiratory distress syndrome
Source: Front Immunol. 2025 Nov 6;16:1620745. doi: 10.3389/fimmu.2025.1620745 (PMC12631193; doi:10.3389/fimmu.2025.1620745)
Supplement: Supplementary file 9 [file Image9.pdf]

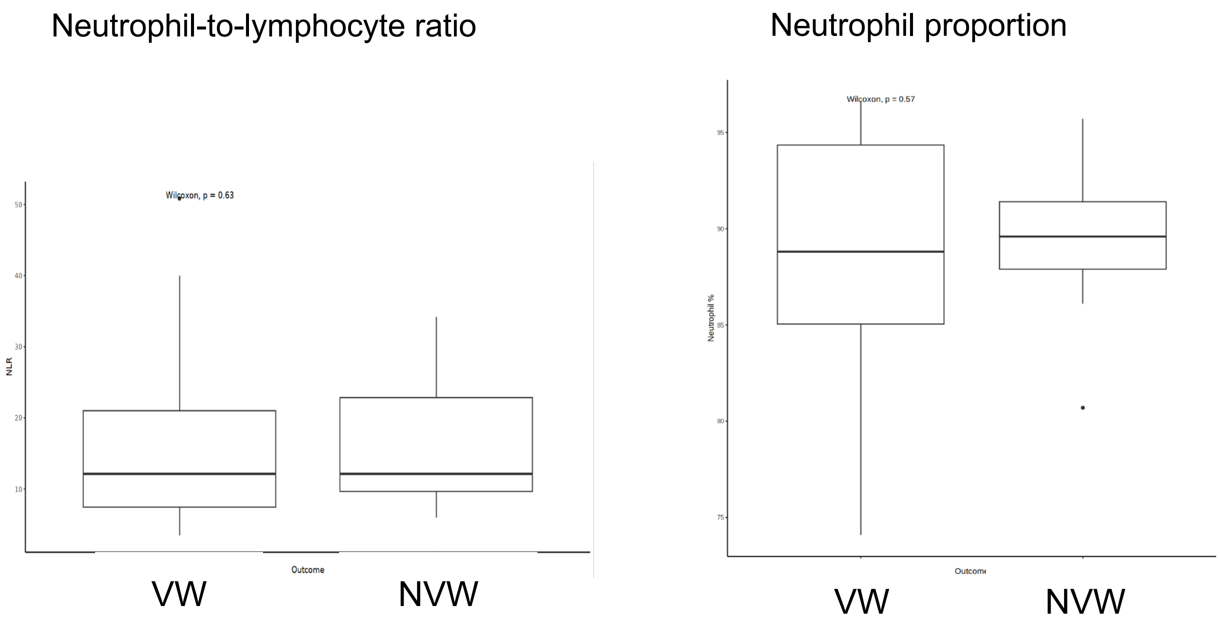

Supplementary Figure 9. Boxplot of neutrophil-to-lymphocyte ratio or neutrophil proportion
